# Fabrication and Characterization of Biomedical Ti-Mg Composites via Spark Plasma Sintering

**DOI:** 10.3390/ma17143470

**Published:** 2024-07-13

**Authors:** Taisei Masuda, Minho Oh, Equo Kobayashi

**Affiliations:** Department of Materials Science and Engineering, School of Materials and Chemical Technology, Tokyo Institute of Technology, S8-18, 2-12-1 Ookayama, Meguro-ku, Tokyo 152-8552, Japanequo@mtl.titech.ac.jp (E.K.)

**Keywords:** spark plasma sintering, Ti-Mg composites, biomaterials, stress shielding, Young’s modulus

## Abstract

The fabrication of Ti-Mg composite biomaterials was investigated using spark plasma sintering (SPS) with varying Mg contents and sintering pressures. The effects of powder mixing, Mg addition, and sintering pressure on the microstructure and mechanical properties of the composite materials were systematically analyzed. Uniform dispersion of Mg within the Ti matrix was achieved, confirming the efficacy of ethanol-assisted ball milling for consistent mixing. The Young’s modulus of the composite materials exhibited a linear decrease with increasing Mg content, with Ti-30vol%Mg and Ti-50vol%Mg demonstrating reduced modulus values compared to pure Ti. Based on density measurements, compression tests, and Young’s modulus results, it was determined that the sinterability of Ti-30vol%Mg saturates at a sintering pressure of approximately 50 MPa. Moreover, our immersion tests in physiological saline underscore the profound significance of our findings. Ti-30vol%Mg maintained compressive strength above that of cortical bone for 6-to-10 days, with mechanical integrity improving under higher sintering pressures. These findings mark a significant leap towards the development of Ti-Mg composite biomaterials with tailored mechanical properties, thereby enhancing biocompatibility and osseointegration for a wide range of biomedical applications.

## 1. Introduction

In recent years, with the increase in demand for medical care due to aging and longevity, there is an expectation for the development of high-functioning and high-value-added biomaterials. Metallic biomaterials are often used as load-bearing components, taking advantage of their high fracture toughness and fatigue strength. Alongside existing biomaterials such as stainless steel (SUS316L), Co-Cr alloys, industrial pure titanium, and titanium alloys, functional metallic biomaterials such as shape memory alloys, gradient function materials, and biodegradable Mg materials have been developed [1,2]. Among these, industrial pure titanium and titanium alloys are widely used in dental and surgical implants due to their high specific strength and excellent corrosion resistance. While the Young’s modulus of titanium alloys (approximately 110 GPa) is significantly smaller compared to other metallic biomaterials like stainless steel or Co-Cr alloys, it is still much larger than the Young’s modulus of cortical bone (7–30 GPa) [2,3,4]. This difference leads to stress shielding, where a significant portion of stress is preferentially borne by the implant, potentially causing an inhibition of bone growth and a decrease in bone density [5]. Therefore, efforts have been made to develop materials such as β-type titanium alloys and porous materials to decrease Young’s modulus [6,7].

It has been found that the Young’s modulus of β-type titanium alloys is lower compared to α-type titanium alloys, and the non-toxic Ti-29Nb-13Ta-4.6Zr (TNTZ) alloy, which exhibits the lowest Young’s modulus of approximately 60 GPa, has been developed [2,8,9]. Additionally, porous materials have a lower apparent Young’s modulus and can enhance osseointegration, where implants bond with living bone at the optical microscope level, allowing for a sufficient fixation of implants [10]. However, there are challenges with β-type titanium alloys, such as the high cost and high melting point of alloying elements like Nb, Ta, Mo, or Zr [11]. On the other hand, porous materials often experience stress concentration in pore regions, leading to inferior mechanical properties, making them more suitable for low-load environments [12].

To address these challenges, research on Ti-Mg composite materials has been conducted [13,14,15]. Magnesium is an essential element in the body and has a modulus of 41 GPa, which is close to the modulus of cortical bone compared to other metallic biomaterials and is cost-effective [16,17]. Since the modulus of composite materials is roughly proportional to the volume fraction, compounding magnesium with a lower modulus than titanium results in Ti-Mg composite materials having a lower modulus than pure titanium and titanium alloys [18,19]. Moreover, when biodegradable magnesium dissolves in the body, the originally magnesium-containing parts transform into pores, changing Ti-Mg composite materials into porous titanium [20,21]. Therefore, Ti-Mg composite materials exhibit properties that initially have superior strength compared to porous titanium due to the presence of magnesium during implantation. As magnesium dissolves during bone recovery and growth, it transforms into porous titanium with low modulus and excellent osseointegration with living bone [22,23,24]. The density and melting point of Ti are 4.506 g/cm^3^ and 1668 °C, respectively, while the density and melting point of Mg are 1.738 g/cm^3^ and 650 °C, respectively. Due to the significant difference in density and melting point, obtaining Ti-Mg composite materials with a uniform structure using conventional casting methods is difficult [25]. Powder metallurgy is practical for manufacturing such metal composite materials with significant differences in properties. Powder metallurgy is a method of producing dense materials by diffusion of metal atoms between powder particles, eliminating the need to melt the metal and allowing for material fabrication at lower temperatures compared to casting methods [26]. In manufacturing Ti-Mg composite materials, the Spark Plasma Sintering (SPS) method has several advantages over other methods [27], such as liquid Mg infiltration, as shown in Table 1, making it suitable for biomedical applications. Specifically, SPS allows for the precise control of sintering temperature and pressure, ensuring uniform microstructural development and enhanced densification. This accurate control minimizes thermal gradients and results in superior mechanical properties. Additionally, SPS significantly reduces processing time compared to liquid infiltration methods and allows for the fine-tuning of microstructural characteristics through adjustable sintering parameters. This flexibility enables tailoring mechanical properties to meet specific biomedical requirements, such as desired Young’s modulus and strength [28].

In this study, Ti-Mg composite materials, which are difficult to manufacture using conventional casting methods, were fabricated using SPS, one of the powder metallurgy techniques. SPS involves the intermittent flow of direct current through the sample, causing discharge between powder particles, heating by joule heating, and pressure application, effectively shortening the sintering time due to rapid heating rates [29,30]. Moreover, materials fabricated using SPS typically exhibit high-density uniform structures, and the short sintering time reduces the likelihood of grain coarsening, resulting in sintered bodies with excellent mechanical properties [28,31]. For the Ti-Mg composite materials produced by changing the composition and sintering pressure, their mechanical properties were evaluated by observing the microstructure, compression tests, and Young’s modulus measurements. How changes in the amount of Mg added and sintering pressure affect the microstructure and mechanical properties was investigated. Additionally, Mg was dissolved by immersing the Ti-Mg composite materials in a physiological saline solution. Mg toxicity typically results from an excessive intake of medications containing magnesium or impaired kidney excretion [32]. Therefore, it is crucial to regulate and monitor its solubility carefully. The compression test was performed on samples immersed for different durations to investigate the mechanical integrity of the composite materials.

## 2. Materials and Methods

Using pure Ti powder (purity 99.98%, spherical, maximum particle size 45 μm) and pure Mg powder (purity 99.5%, irregular shape, average particle size 180 μm) as raw materials, three types of powder were prepared: pure Ti, Ti-30vol%Mg, and Ti-50vol%Mg. Pure Ti remained pure Ti powder, while a pulverizing ball mill (Pulverisette 7 classicline, Fritsch, Germany) was used to mix the composite powder. A stainless-steel mixing container was filled with 50 wt% ZrO_2_ balls with a diameter of 1 mm and 5 wt% ethanol as the mixing solvent. After mixing for 5 min at 500 rpm, the rotation was stopped for 5 min, then reversed, and mixing was continued for another 5 min at 500 rpm. After mixing, ZrO_2_ balls were separated from the mixed powder using a 600 μm sieve. The purpose of the ball mill in this study is to prevent powder aggregation by solvent and aim for a more uniform mixture rather than particle crushing. The powder-mixing operation was conducted in a glove box filled with Ar gas. Before the sintering process, X-ray diffraction (XRD, MiniFlex600 by Rigaku, Tokyo, Japan) analysis was performed with angle conditions set at 20°–90° and a step size of 0.01° on three types of powders (pure Ti powder, pure Mg powder, and Ti-30Mg mixed powder), to verify their composition and crystallinity [33,34]. Carbon paper with a thickness of 0.2 mm was placed on the bottom and sides of the graphite die (NJS-Japan, Tokyo, Japan), and each powder was filled into the graphite die inside a glove box. The powders were then compressed using a hydraulic pump at 20 MPa for 1 min to obtain a compacted body. The sintering container, now containing the compacted body, was carefully installed in the chamber of the SPS device (511S, SPS Syntex, Tokyo, Japan). The vacuum level inside the chamber was meticulously maintained at 50 Pa. The sintering temperature, a critical parameter that directly influences the final properties of the sintered samples, was set to 580 °C. Similarly, the sintering pressure, another key parameter that significantly affects the sintering process, was set to 25, 50, 75, and 100 MPa, respectively. These settings were crucial to achieving the desired sintering results. After sintering, the samples were unloaded without waiting for cooling and cooled while maintaining the vacuum inside the chamber. Table 2 shows the Mg content and sintering conditions of the fabricated samples.

The sintered specimen was cut using a microcutting system (Accustom-5, Struers, Tokyo, Japan), and the cross-section was polished with #500 to #4000 SiC emery papers, followed by polishing with 3 μm diamond spray as the abrasive. After ultrasonic cleaning with isopropanol for 5 min., observations were made using an optical microscope (OM) (DMI3000M, Leica Microsystems, Wetzlar, Germany) and field emission scanning electron microscopy (FESEM) (JSM-7200F, JEOL, Tokyo, Japan), and an elemental analysis and element distribution map (EDM) were performed using energy dispersive spectroscopy (EDS) (JED-2300 Analysis Station Plus, JEOL, Tokyo, Japan). The powder, fracture surface, and post-infiltration structure were also observed using FESEM without polishing.

The sintered specimens were cut into dimensions of 4 mm × 4 mm × 8 mm and polished using #1000 SiC emery paper. The vertical, horizontal, and height dimensions of the samples were measured three times using a micrometer, and the average values were used to determine the dimensions. The weight of each sample was measured to calculate its density. Compression tests were conducted using an Autograph universal testing machine (AG-1 1000 kN, Shimadzu, Tokyo, Japan). Nominal strain and nominal stress were calculated using the above measurement values in the compression tests. The tests were performed three times for each specimen, and stress–strain curves were plotted based on the results. To measure Young’s modulus, an ultrasonic pulse velocity test was conducted. This is a non-destructive technique that involves propagating ultrasonic pulses through the sample using longitudinal and transverse wave transducers. Young’s modulus was then determined based on the velocity of the ultrasonic pulses.

Using SiC emery paper ranging from #500 to #4000, the bottom of the sintered specimens was polished to be parallel, then polished with a diamond spray (3 μm). After preparing cylindrical specimens with a diameter of approximately 20 mm and a thickness of about 9 mm, the thickness of the samples was measured at seven points using a micrometer, and the average of five data points, excluding the maximum and minimum values, was taken as the thickness of the sample. For the measurements, an ultrasonic flaw detector (USM35X, GE Measurement and Control, MA, USA), longitudinal wave probe (G5KB, GE Measurement and Control, MA, USA), and transverse wave probe (B2C10SN, ITeS Corporation, Tokyo, Japan) were used. Measurements were conducted seven times for both longitudinal and transverse waves, and the average of five data points, excluding the maximum and minimum values, was taken as the velocity to calculate Young’s modulus. Due to the insufficient size of the immersion test samples, a unique approach was taken to calculate Young’s modulus [35,36]. The slope in the elastic region of the stress–strain curve obtained from the compression test was used. However, it is important to note that the Young’s modulus calculated from this slope in the compression test shows lower values than the usual Young’s modulus. Therefore, the evaluation of the Young’s modulus change according to the immersion time was conducted by setting 0 days of immersion as 100% and evaluating the decrease rate of Young’s modulus.

For the immersion test, two types of sintered materials were prepared for compression testing and observation, respectively, and each sintered material was cut into rectangular shapes of 4 mm × 4 mm × 8 mm. The sides were polished with #1000 SiC emery paper, and then the height, width, and length were each measured three times using a micrometer. Finally, ultrasonic cleaning was conducted for 300 s using isopropanol. A physiological saline solution was prepared by adding 18 g of sodium chloride to 2 L of distilled water. The specimens were immersed in 50 mL of physiological saline solution per 10 mm^2^ of sample surface area, and the temperature was maintained at 37 °C using a muffle furnace (FO-60P, Glass Kiki Co., Ltd., Tokyo, Japan) [37]. After the immersion test, the samples were washed with distilled water for 60 s, followed by compression testing. Additionally, the observation samples were immersed in a mixture of chromic acid and nitric acid for 60 s to remove corrosion products, then they were washed with distilled water before observation.

## 3. Results and Discussion

### 3.1. Evaluation of Powders

**Figure 1:**
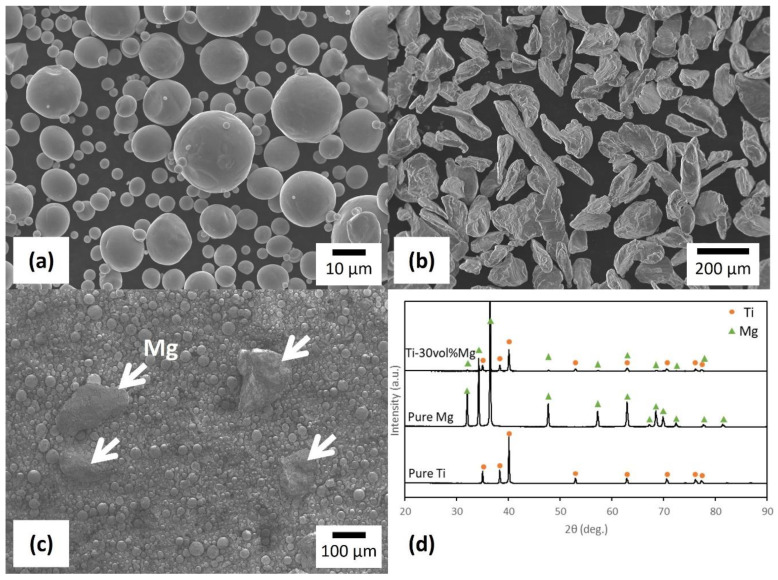
FESEM micrographs of (**a**) Ti powder, (**b**) Mg powder, and (**c**) the Ti-30Mg mixed powder obtained from (**a**,**b**). (**d**) XRD patterns of the powders.

Figure 1a,b, respectively, show the appearance of Ti and Mg powders observed through FESEM. The appearance of the Ti-30Mg mixed powder obtained by mixing this raw material powder is shown in Figure 1c. The portion indicated by the white arrows in this figure is Mg particles, and it was observed that large and irregularly shaped Mg particles are dispersed within the relatively small spherical Ti powder. Mechanical mixing was performed using a ball mill. However, there was no significant difference in the particle size of the Mg particles compared to that of the Mg raw material powder. Due to the short mixing time of 10 min, most of the Mg particles appear to have remained uncrushed. Generally, the effective diameter of pores for bone cells to penetrate and proliferate within pores in porous biomaterials is reported to be between 100 μm and 400 μm [38,39]. Since the average size of the Mg particles remained uncrushed at 180 μm in this mixing condition, good osteoinductivity of the porous body after Mg dissolution can be expected.

Additionally, the analysis of the Mg particle surfaces in the mixed powder revealed smooth surfaces. However, the Mg particles, after mixing, were found to be covered with fine attachments. The EDS point analysis of these attachments mostly revealed pure Mg, while in some white attachments, an oxygen concentration of 53.41 at% was determined, indicating magnesium oxide. The temperature of the mixing vessel rises due to collisions between the vessel walls, ZrO_2_ balls, and the raw material powder in the ball mill, promoting the reaction between ethanol and Mg particles and resulting in the formation of compounds. Figure 1d represents the XRD analysis results for the powder. No peaks other than Ti and Mg were observed in pure Ti and pure Mg. Although peaks of Ti and Mg were observed simultaneously in Ti-30Mg, peaks of oxides were not observed. Although the presence of reaction products was identified through FESEM and EDS analysis, they were only present on the surface of Mg particles and in small amounts overall. Hence, no peaks were observed.

### 3.2. Microstructure of Sintered Composites

**Figure 2:**
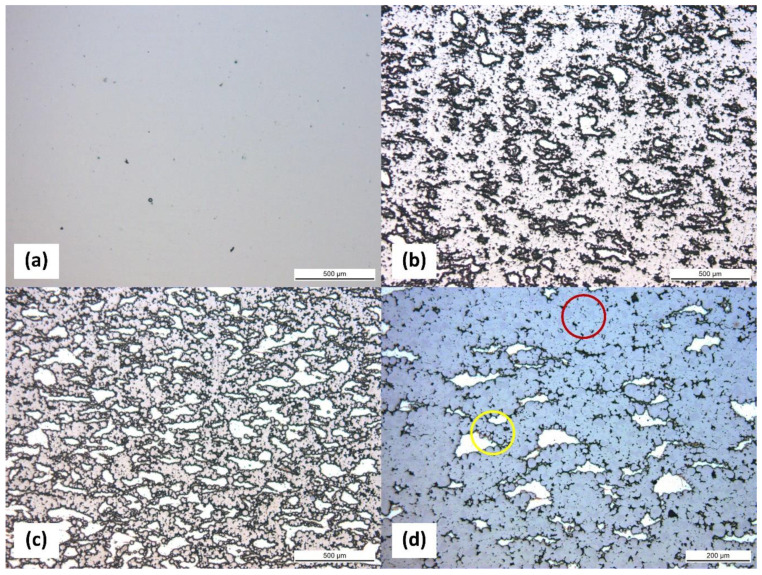
Optical micrographs of (**a**) Pure Ti, (**b**) Ti-30Mg, and (**c**) Ti-50Mg samples. (**d**) Enlarged optical micrographs of Ti-30Mg.

Figure 2a–c depict micrographs of Pure Ti, Ti-30Mg, and Ti-50Mg by optical microscope, respectively. The vertical direction represents the compression direction during sintering. Although 580 °C is a relatively low sintering temperature for Ti, the result of sintering with Pure Ti showed only a small amount of porosity. According to density measurements, the relative density was 91%, meaning the porosity was 9%, indicating sufficient densification of Pure Ti even at 580 °C, as shown in Table 3. With a sintering pressure of 50 MPa, it can be observed that Mg is uniformly dispersed within the Ti matrix in Ti-30Mg and Ti-50Mg. Mg particles appear slightly flattened perpendicular to the compression direction, indicating deformation due to sintering pressure. Based on the calculation of porosity using relative density, the addition of Mg resulted in a decrease in porosity due to its effect of filling the pores between Ti particles. The lowest pressure sample, Ti-30Mg (TM25), exhibited a porosity of 6.4%, while TM50-TM100 showed results close to 0%.

Furthermore, an increase in porosity within the Ti matrix compared to Pure Ti can be observed. Figure 2d presents an enlarged image of the microstructure of Ti-30Mg, where, as indicated by the red circle, the regions without Mg show well-progressed sintering with neck growth between Ti particles, whereas, as noted in the yellow circle, Ti particles near the Mg particles do not exhibit neck growth and maintain the shape of the raw powder. This suggests that insufficient sintering stress is applied to Ti particles near Mg particles due to Mg deformation, resulting in delayed sintering progress. The increase in porosity within the Ti matrix is also presumed to be due to the deformation of Mg during sintering. Various parameters affect the structure and mechanical properties in the production of specimens through powder sintering, such as composition, powder particle size, powder mixing method, and sintering conditions. Especially in the case of discharge plasma sintering, the sintering conditions vary, and changes in temperature, holding time, heating rate, pressure, discharge pulse interval, electric current, etc., alter the sintering characteristics. In this study, the sintering temperature was limited to a maximum of 650 °C to conduct sintering at a temperature where Mg does not dissolve. Therefore, to enhance the sintering characteristics of Ti particles, the effect of sintering pressure on the sintering properties of Ti-30vol%Mg composition was investigated in the present study.

**Figure 3:**
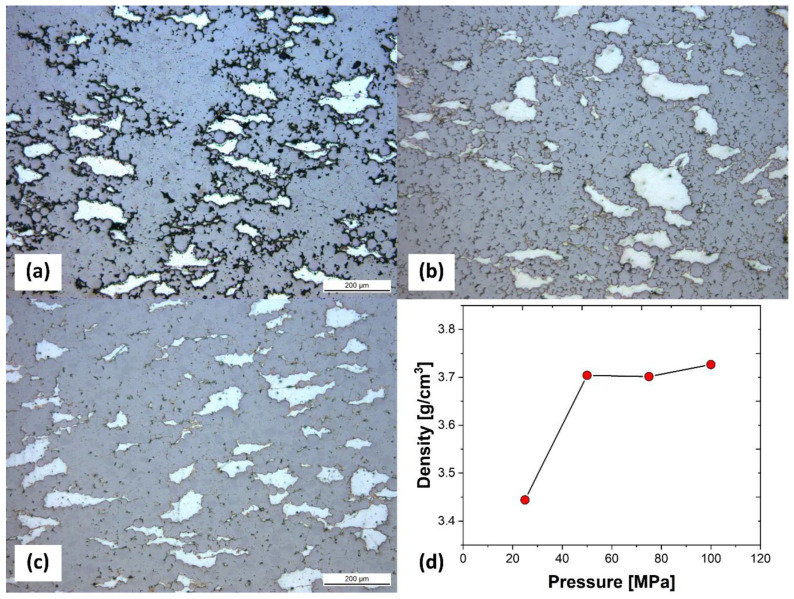
Optical micrographs of Ti-30vol%Mg sintered at (**a**) 25 MPa (TM25), (**b**) 75 MPa (TM75), and (**c**)100 MPa (TM100). (**d**) Relationship between sintering pressure and density.

Figure 3a–c depict the microstructure of specimens TM25, TM75, and TM100, respectively, which were sintered under different pressures of 25, 75, and 100 MPa, using Ti-30vol%Mg mixed powder raw materials. TM100 sintered at the highest pressure of 100 MPa showed fewer pores within the Ti phase. Through the microstructure, it was possible to confirm that sinterability improves with increasing sintering pressure. To quantify sinterability, the density of each specimen was measured, and density changes with pressure variations were represented, as shown in Figure 3d. The density of TM25 showed a smaller value (3.44 g/cm^3^) compared to other specimens, indicating the presence of many pores within the specimen. While the density increased significantly up to 3.70 g/cm^3^ in TM50, there was only a slight increasing trend in TM50, TM75, and TM100, with no significant difference in density. When manufacturing Ti-Mg composites using SPS, the porosity of the composite material can be influenced by several factors, in addition to sintering pressure, such as sintering temperature, Mg composition, and the size and shape of the powders [15,40]. Specifically, as the Mg composition increases, the diffusion of Mg, which has a relatively lower melting point, occurs rapidly, leading to a decrease in porosity and an increase in density [15,41].

The sintering process consolidates powder particles into a solid mass by applying heat and pressure. Several mechanisms govern this process, including powder particle interactions, diffusion kinetics, and microstructural evolution [42]. During sintering, powder particles come into contact with each other due to applied pressure. At the contact points, known as necks, atomic bonds form between particles, facilitating the consolidation process. The initial stage involves surface diffusion, where atoms migrate along the particle surfaces to form bonds [43,44,45,46]. As sintering progresses, bulk diffusion becomes dominant, with atoms diffusing through the lattice of particles to further densify the material. Diffusion plays a critical role in sintering, as it governs the movement of atoms within the powder compact. The diffusion rate depends on factors such as temperature, pressure, and the chemical composition of the powder [43]. High temperatures increase atomic mobility, promoting faster diffusion and densification. Pressure reduces the activation energy required for diffusion, accelerating the sintering process. Additionally, the chemical composition of the powder influences the diffusion kinetics, as different elements diffuse at varying rates [47]. As sintering progresses, the microstructure of the material undergoes significant changes. Initially, pores between powder particles are eliminated as necks form and grow. As sintering continues, the pores decrease in size and number, increasing material density. The microstructure evolves from a network of interconnected pores to a solid, dense structure. However, excessive sintering can result in grain growth and the formation of large pores, negatively impacting the mechanical properties of the material. The microstructural analysis revealed a uniform dispersion of Mg within the Ti matrix, which was achieved through mechanical alloying and spark plasma sintering. This uniform distribution is critical for ensuring consistent mechanical properties throughout the composite. The formation of fine Ti-Mg intermetallic phases suggests successful bonding between the titanium and magnesium particles, which is crucial for enhancing the composite’s mechanical performance. The presence of these intermetallic phases can be attributed to the high heating rates and localized temperature spikes inherent in the spark plasma sintering process, promoting rapid diffusion and reaction between Ti and Mg.

### 3.3. Mechanical Properties Depending on Mg Contents

**Figure 4:**
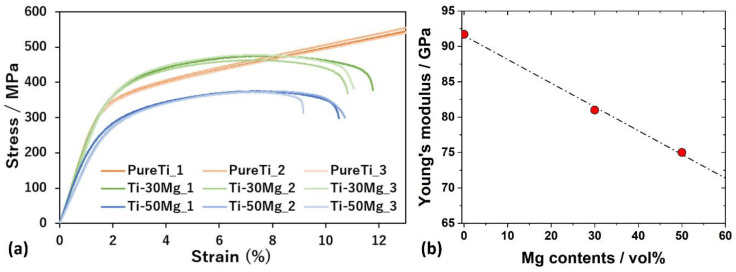
(**a**) Compressive stress–strain curves of pure Ti, Ti-30Mg, and Ti-50Mg. (**b**) Relationship between Mg content and Young’s modulus.

Figure 4a shows the stress–strain curves obtained from compression tests of Pure Ti, Ti-30Mg, and Ti-50Mg. In the compression test of Pure Ti, the specimen did not fracture, and the stress continued to increase, so the test was stopped at the point exceeding the fracture strain of Ti-30Mg and Ti-50Mg. The compressive strength of cortical bone is estimated to be approximately 180 MPa, and all specimens exhibited higher strengths than cortical bone. When comparing the stress–strain curves of pure Ti, where Mg compounds are not present in the powder, and Ti-30Mg and Ti-50Mg, where Mg compounds are formed on the surface of Mg, no significant decrease in mechanical properties due to compounds in the powder was observed. The yield stresses were 311 MPa for Pure Ti, 334 MPa for Ti-30Mg, and 239 MPa for Ti-50Mg, with Ti-30Mg showing a higher value than pure Ti. This is believed to be due to the solid solution of oxygen in Ti. According to the Ti-O binary phase diagram, the solubility limit of oxygen in Ti is approximately 33% at both sintering and room temperatures, which is significantly high. It is considered that oxygen solubility occurred due to exposure of the powder to air during transportation until installation in the chamber of the SPS device, as well as oxygen atoms from ethanol, the mixing solvent. On the other hand, the yield stress of Ti-50Mg was lower than that of Pure Ti and Ti-30Mg. This is attributed to a more significant decrease in strength due to a reduction in the volume fraction of Ti than to solid solution strengthening by oxygen in Ti. Figure 4b illustrates the relationship between Mg content and Young’s modulus measured by the ultrasonic pulse method. Meanwhile, the Young’s modulus of typical pure Ti is 106 GPa, and that of pure Ti is 92 GPa, which is believed to be due to the presence of 9% porosity in the sintered composite specimen. The Young’s modulus of Ti-30Mg and Ti-50Mg were 81 GPa and 75 GPa, respectively, confirming that the Young’s modulus decreases with increased Mg content.

The microstructure and phase composition can significantly affect the stress–strain response observed in compression tests of Ti-Mg composites. The microstructure influences the load-bearing capability and deformation mechanisms of the composite [48,49]. A well-sintered microstructure with fewer pores and a uniform distribution of Mg within the Ti matrix tends to exhibit better mechanical properties. Pores act as stress concentrators and can initiate cracks, reducing the material’s strength and ductility [50]. The content of Mg in the composite has a direct effect on Young’s modulus due to the lower modulus of Mg compared to Ti. Generally, as the Mg content increases, the overall modulus of the composite decreases. This is because the modulus of composite materials is roughly proportional to the volume fraction of the constituent phases [51]. Therefore, with a higher volume fraction of Mg (which has a modulus of 41 GPa) compared to Ti (which has a modulus of about 110 GPa), the composite’s modulus will be lower than pure Ti.

**Figure 5:**
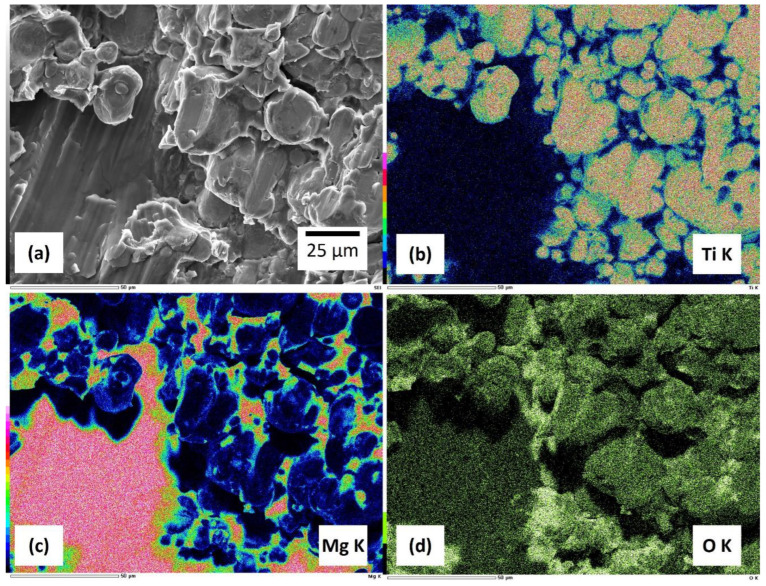
(**a**) Fractography of Ti-30Mg by FESEM. Element distribution maps of (**b**) Ti, (**c**) Mg, (**d**) O by EDS.

Figure 5 depicts SEM images of the fracture surface after compression testing of Ti-30Mg and the results of element distribution mapping by EDS analysis. The fracture surface of the Mg portion exhibits a river line crack pattern, indicating brittle fracture in the Mg portion. In contrast, the Ti portion maintains the shape of the raw powder, suggesting that the fracture in the Ti portion mainly occurred at the particle boundaries of the powder. From these observations, it can be understood that atomic diffusion at the Ti particle boundaries did not sufficiently progress. Optimizing the sintering conditions is expected to enhance the overall mechanical properties of the composite material by improving the sinterability of Ti [52].

**Figure 6:**
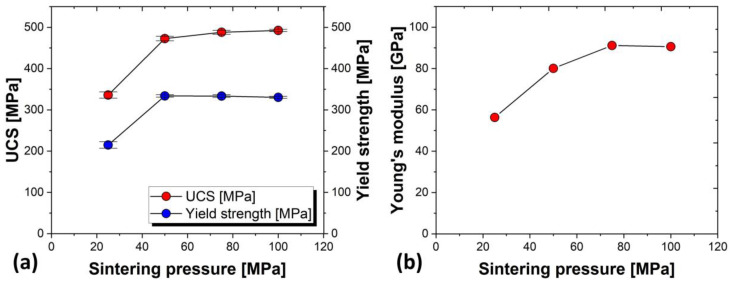
(**a**) Changes in UCS and Yield strength with variations in sintering pressure. (**b**) Relationship between sintering pressure and Young’s modulus.

Figure 6a plots the changes in uniaxial compressive strength (UCS) and yield strength with variations in sintering pressure, obtained from the stress–strain curves of TM25, TM50, TM75, and TM100 from the compression test results. Only TM25 showed distinctly different compression test results compared to other specimens, with minimum compressive strength and yield strength. TM50, TM75, and TM100 showed no significant differences in compression behavior, compressive strength, or yield strength, similar to density. Additionally, Figure 6b presents the results of Young’s modulus measurements at each sintering pressure. Young’s modulus increased with increasing sintering pressure, from 57 GPa in TM25 to 91 GPa in TM75 and TM100. Since this study aims to produce Ti-Mg composite materials with low Young’s modulus, a lower modulus is desirable. However, considering that Mg dissolves during insertion into the body, leading to porous Ti, and prioritizing the sinterability of the matrix Ti, an increase in Young’s modulus due to increased sintering pressure is considered a favorable outcome. Since only TM25 showed significantly lower values in density measurements, and there were no significant differences in TM50, TM75, and TM100, it can be assumed that the sinterability of Ti-30vol%Mg saturates around 50 MPa.

The significant effect of sintering pressure on Young’s modulus, compared to UCS or yield strength, can be attributed to the microstructural changes that occur during the sintering process. Higher sintering pressures lead to better densification of the material, reducing porosity and increasing the contact area between grains [28]. This improved grain boundary contact enhances the material’s ability to resist deformation, thereby increasing Young’s modulus. Sintering pressure helps in achieving a more homogeneous microstructure. A uniform microstructure with fewer defects and voids contributes to a higher Young’s modulus because the material can deform elastically more uniformly under stress. Young’s modulus is more sensitive to changes in microstructure and density than UCS or yield strength [53]. While UCS and yield strength are influenced by factors like grain size and the presence of flaws, Young’s modulus is directly related to the stiffness of the material, which is significantly affected by the degree of densification and the quality of grain boundaries. During sintering, pressure aids in the formation of stronger bonds and larger necks between particles. These stronger inter-particle bonds contribute to a higher elastic modulus, as the material can better resist elastic deformation.

The Mg content and the sintering parameters significantly influence the mechanical properties of the Ti-Mg composites. The addition of Mg reduces the overall density of the composites, making them lighter than pure titanium. This reduction in density is advantageous for biomedical implants, as it can lead to less stress on surrounding bone and tissue. The Young’s modulus of the composites can be tailored by adjusting the Mg content. Composites with 5–15 wt% Mg exhibited Young’s modulus closer to that of natural bone, which is beneficial in minimizing stress-shielding effects. This characteristic addresses a significant drawback of conventional titanium implants, which have a much higher modulus than bone, leading to stress shielding and bone resorption over time. The compressive strength of the composites decreases with increasing Mg content. However, the values obtained are still within acceptable ranges for load-bearing applications. This trade-off between modulus and strength must be carefully balanced to optimize implant performance.

### 3.4. Immersion Test in Physiological Saline

**Figure 7:**
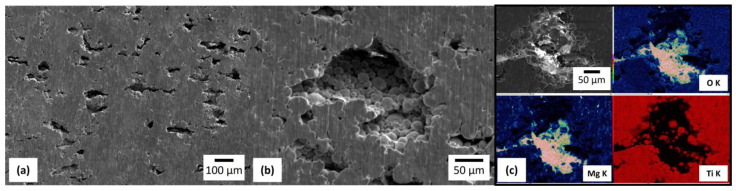
(**a**) A FESEM micrograph of the surface of the Ti-30Mg sample, (**b**) a magnified micrograph for pore observation after 1 day of immersion in saline solution, (**c**) Element distribution maps of O, Mg, and Ti by EDS.

Figure 7 shows the microstructure of Ti-30Mg after immersing in physiological saline for 1 day. The observation was conducted after immersion for 1 min in a mixed solution of chromic acid (H_2_CrO_4_) and silver nitrate (AgNO_3_) to remove corrosion products. In Figure 7a, the dissolution of Mg and the formation of pores on the sample surface were observed. The micrograph in Figure 7b, magnified inside the pores, shows that most Ti particles maintain the shape of the raw powder. Here, as in the cross-sectional micrograph of OM, it can be observed that the sintering of Ti particles around the Mg powder was not fully completed. Figure 7c shows the element distribution maps of the pore region after the dissolution of Mg. Mg was detected on the pore’s surface, and oxygen was distributed along with Mg.

**Figure 8:**
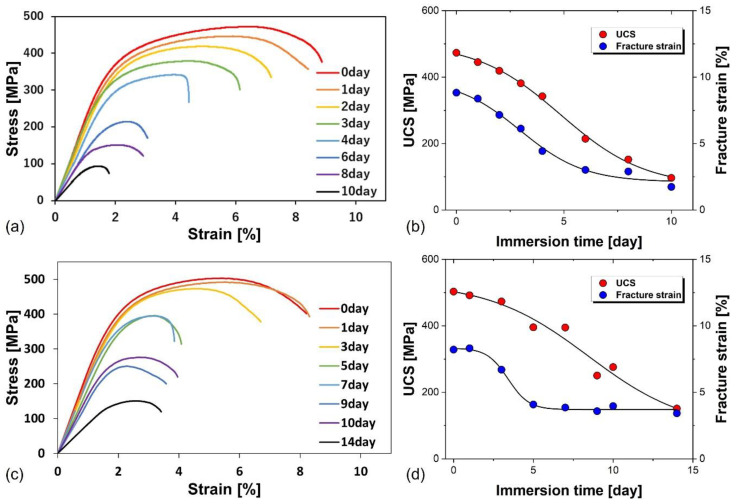
Compressive stress–strain curves of Ti-30Mg samples with (**a**) 50 MPa and (**c**) 100 MPa of sintering pressures immersed in saline for various times up to 10 days. Changes of UCS and fracture strain of Ti-30Mg with (**b**) 50 MPa and (**d**) 100 MPa of sintering pressures increasing immersion times in saline for up to 10 days.

**Figure 9:**
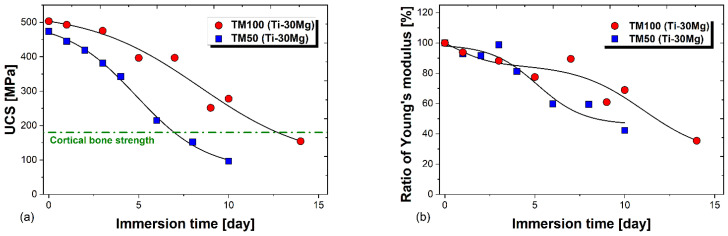
(**a**) Relationship between UCS and immersion time of TM100 (Ti-30Mg) and TM50 (Ti-30Mg). (**b**) Changes in the ratio of Young’s modulus to the initial Young’s modulus with an increase of the immersion time for TM100 (Ti-30Mg) and TM50 (Ti-30Mg).

Figure 8a presents the stress–strain curves of compression tests after immersion tests for various durations. At the same time, Figure 8b shows the changes in UCS and fracture strain according to immersion time. After 6 days of immersion, the compression strength was 214 MPa, and Ti-30Mg could maintain a strength higher than that of cortical bone (approximately 180 MPa [54]) until 6 days after immersion. Both compression strength and fracture strain decreased with increasing immersion time. UCS and fracture strain showed nonlinear deceleration with immersion time. In both cases, it was observed that the experimental results closely matched a sigmoidal curve based on the Boltzmann function. Figure 8c shows the stress–strain curves obtained from compression tests of TM100 (Ti-30Mg) fabricated with a sintering pressure of 100 MPa for various immersion times. At the same time, Figure 8d illustrates the changes in compressive strength and fracture strain during immersion. Similar to the compression test results for TM50 (Ti-30Mg) in Figure 8b, the UCS and fracture strain decreased with increasing immersion time due to the dissolution of Mg within the composite material. The UCS on the 9th and 10th days of immersion were 250 MPa and 276 MPa, respectively, maintaining strengths exceeding those of cortical bone until the 10th day. Figure 9a plots the variation in UCS with increasing immersion time for both TM50 (Ti-30Mg) and TM100 (Ti-30Mg), with a green dash-dot horizontal line representing cortical bone strength. It can be inferred that the mechanical integrity improves with increasing sintering pressure, as TM100 maintains strength above that of cortical bone for a longer duration compared to TM50.

Mechanical property changes were relatively significant in the early stages of immersion. However, as time increased, the changes became relatively small, and there was almost no change in fracture strain after the 6th day. This is believed to be because Mg dissolved from the entire sample surface during the early stages of immersion, but subsequently, dissolution of Mg from the center of the sample was required. The dissolution of Mg occurs through the chemical reactions below [55], resulting in the generation of hydrogen gas and corrosion products where Mg is dissolved.

Mg + 2H_2_O = Mg(OH)_2_ + H_2_

Mg(OH)_2_ + 2Cl− = MgCl_2_ + 2OH^−^

When hydrogen gas or corrosion products are generated inside the pores, it takes time for the solution to penetrate into the center of the specimen. Therefore, as the immersion time passes, it is expected that mechanical property changes will diminish, especially after a certain period has elapsed.

At the outset, the immersion phase is crucial as it sets the stage for the corrosion process. Here, the Mg in the Ti-Mg composite swiftly interacts with the saline solution, leading to the creation of Mg(OH)_2_ and hydrogen gas [56,57]. These reactions initiate the formation of a porous Mg(OH)_2_ layer on the surface, which initially provides some defense but gradually transforms into more soluble MgCl_2_ in the presence of chloride ions from the saline solution. As immersion continues, the protective Mg(OH)_2_ layer gradually breaks down, releasing Mg ions into the solution. The generated hydrogen gas can form bubbles at the interface, further promoting Mg degradation [19,58]. This stage is characterized by significantly reducing mechanical properties as Mg content diminishes. Over time, the corrosion rate slows down as the more easily accessible Mg is depleted, and the remaining Mg is less exposed due to deeper penetration into the composite. The changes in mechanical properties, mainly compressive strength, decrease at this stage [18]. Our immersion tests have yielded significant findings. Ti-30vol%Mg maintained its compressive strength above that of cortical bone for a considerable period of up to 10 days. This behavior suggests that the composite can retain its mechanical integrity for a sufficient duration, potentially supporting the initial bone healing processes. The release of Mg ions plays a critical role in the biodegradation process. Mg ions are known to be biocompatible and can enhance osteogenesis. However, the localized increase in pH due to the formation of OH^−^ ions needs to be managed to prevent adverse effects on surrounding tissues.

Figure 9b illustrates the variation in Young’s modulus during immersion for specimens of TM50 (Ti-30Mg) and TM100 (Ti-30Mg). Here, the reduction in Young’s modulus was evaluated with respect to the specimen at 0 days before immersion, which was considered 100%. It can be observed that Young’s modulus decreases with increasing immersion time for both specimens. For TM50 (Ti-30Mg), Young’s modulus decreased by 40% after 6 days of immersion compared to before immersion, resulting in a modulus of approximately 49 GPa, considering that the modulus of Ti-30Mg was measured to be 81 GPa. In the case of TM100 (Ti-30Mg), the modulus decreased overall by 31% after 10 days of immersion, indicating a modulus of approximately 63 GPa at Day 10 of immersion, considering that the modulus of TM100 measured by ultrasonic pulse method was 91 GPa. It was observed that sintering pressure enhancement led to improved sinterability and mechanical properties. However, in cases where the specimens maintained strengths above that of cortical bone (6 days for TM50 and 10 days for TM100), the modulus after extended immersion was higher for TM100 than TM50. To suppress stress shielding, it is ideal for the modulus after immersion to be as small as possible and closer to that of cortical bone. By adjusting multiple conditions, such as increasing sintering pressure to enhance sinterability and increasing Mg content to reduce the modulus, it is possible to produce Ti-Mg composite materials with higher strength and lower modulus.

## 4. Conclusions

Uniform Ti-Mg composite materials were fabricated by mixing pure Ti powder with low-modulus and biodegradable Mg powder using spark plasma sintering. The effects of mixing powder, evaluating Ti-Mg composite materials, and the influence of Mg addition and sintering pressure on the properties of Ti-Mg composite materials were investigated systematically.

1. The uniform dispersion of Mg in Ti-30Mg and Ti-50Mg and the confirmation of the effectiveness of powder mixing with ethanol-added ball milling in producing uniform Ti-Mg composite materials further solidify the reliability of our findings.
2. Young’s modulus of pure Ti, Ti-30Mg, and Ti-50Mg is 92 GPa, 81 GPa, and 75 GPa, respectively. This indicates a linear decrease in Young’s modulus of Ti-Mg composite materials with Mg addition.
3. From density measurement, compression tests, and Young’s modulus measurement results, only TM25 (Ti-30Mg) showed significantly smaller values, while TM50 (Ti-30Mg), TM75 (Ti-30Mg), and TM100 (Ti-30Mg) showed no significant differences. Therefore, the sinterability of Ti-30vol%Mg saturates around a sintering pressure of approximately 50 MPa.
4. TM50 (Ti-30Mg) showed a decrease in Young’s modulus from 81 GPa to 49 GPa after 6 days of immersion, while TM100 (Ti-30Mg) showed a decline from 91 GPa to 63 GPa after 10 days of immersion, indicating a reduction in stress shielding phenomenon.
5. During immersion in physiological saline, TM50 maintained compressive strength above cortical bone for 6 days and TM100 for 10 days. This confirms that the mechanical integrity of Ti-30vol%Mg improves with increasing sintering pressure.

## Figures and Tables

**Table 1:** Comparison between spark plasma sintering (SPS) and liquid Mg-infiltration methods.

Feature	Spark Plasma Sintering (SPS)	Liquid Mg Infiltration
Temperature Control	Precise, localized heating reduces thermal gradients.	High temperatures required for Mg melting (>650 °C).
Reaction Time	Short sintering times due to efficient heating and pressure.	Longer processing times are needed for complete infiltration.
Microstructure Control	Fine control over microstructure through adjustable parameters.	Dependent on porous Ti structure and Mg flow dynamics.
Porosity Control	Ability to produce dense and controlled porosity composites.	Porosity depends on the initial Ti structure.
Material Homogeneity	Uniform distribution of Mg within Ti matrix.	Potential for uneven Mg distribution.
Mechanical Properties	Tunable properties through precise control of sintering parameters.	Limited by the inherent properties of porous Ti and Mg.
Scalability	Suitable for small-to-medium-scale production.	It can be challenging for large-scale uniform production.
Equipment and Cost	Requires SPS equipment, potentially high initial cost.	Lower equipment cost but higher operational complexity.
Post-processing Needs	Minimal, often no additional machining is required.	It may require additional machining to achieve the final shape.
Applications	Ideal for biomedical implants with tailored properties.	Suitable for large implants where high porosity is needed.

**Table 2:** Specimen conditions for Mg content and sintering pressure.

Specimen	Mg Content (Vol%)	Sintering Pressure (MPa)
Pure Ti	0	50
Ti-30Mg	30	50
Ti-50Mg	50	50
Ti-30Mg (TM25)	30	25
Ti-30Mg (TM75)	30	75
Ti-30Mg (TM100)	30	100

**Table 3:** Porosity data after sample preparation by SPS.

Specimen	Porosity of Sample (%)
Pure Ti	9.0
Ti-30Mg	0
Ti-50Mg	0
Ti-30Mg (TM25)	6.4
Ti-30Mg (TM75)	0
Ti-30Mg (TM100)	0

## Data Availability

The raw data supporting the conclusions of this article will be made available by the authors on request.
